# A specific immune signature for predicting the prognosis of glioma patients with IDH1-mutation and guiding immune checkpoint blockade therapy

**DOI:** 10.3389/fimmu.2022.1001381

**Published:** 2022-09-09

**Authors:** Zhirui Zeng, Chujiao Hu, Wanyuan Ruan, Jinjuan Zhang, Shan Lei, Yushi Yang, Pailan Peng, Feng Pan, Tengxiang Chen

**Affiliations:** ^1^ Transformation Engineering Research Center of Chronic Disease Diagnosis and Treatment, Guizhou Medical University, Guiyang, China; ^2^ Department of Physiology, School of Basic Medicine, Guizhou Medical University, Guiyang, China; ^3^ State Key Laboratory of Functions and Applications of Medicinal Plants, Guizhou Medical University, Guiyang, China; ^4^ Guizhou Provincial Engineering Technology Research Center for Chemical Drug R&D, Guizhou Medical University, Guiyang, China; ^5^ School of Clinical Medicine, Guizhou Medical University, Guiyang, China; ^6^ Department of Pathology, Affiliated Hospital of Guizhou Medical University, Guiyang, China; ^7^ Department of Gastroenterology, Affiliated Hospital of Guizhou Medical University, Guiyang, China; ^8^ Department of Bone and Joint Surgery, Gui Zhou Orthopedic Hospital, Guiyang, China

**Keywords:** immune, signature, glioma, IDH1 mutation, immune checkpoint blockade therapy

## Abstract

Isocitrate dehydrogenase (IDH1) is frequently mutated in glioma tissues, and this mutation mediates specific tumor-promoting mechanisms in glioma cells. We aimed to identify specific immune biomarkers for IDH1-mutation (IDH1mt) glioma. The Cancer Genome Atlas (TCGA) and Chinese Glioma Genome Atlas (CGGA) were used to obtain RNA sequencing data and clinical characteristics of glioma tissues, while the stromal and immune scores of TCGA glioma tissues were determined using the ESTIMATE algorithm. Differentially expressed genes (DEGs), the protein–protein interaction(PPI) network, and least absolute shrinkage and selection operator (LASSO) and Cox regression analyses were used to select hub genes associated with stroma and immune scores and the prognoses of patients and to construct the risk model. The practicability and specificity of the risk model in both IDH1mt and IDH1-wildtype (wtIDH1) gliomas in TCGA and CGGA were evaluated. Molecular mechanisms, immunological characteristics and benefits of immune checkpoint blockade therapy in glioma tissues with IDH1mt were analyzed using GSEA, immunohistochemical staining, CIBERSORT, and T-cell dysfunction and exclusion (TIDE) analysis. The overall survival rate for IDH1mt-glioma patients with high stroma/immune scores was lower than that for those with low stroma/immune scores. A total of 222 DEGs were identified in IDH1mt glioma tissues with high stroma/immune scores. Among them, 72 genes had interactions in the PPI network, while three genes, *HLA-DQA2*, *HOXA3*, and *SAA2*, were selected as hub genes and used to construct risk models classifying patients into high- and low-risk score groups, followed by LASSO and Cox regression analyses. This risk model showed prognostic value in IDH1mt glioma in both TCGA and CCGA; nevertheless, the model was not suitable for wtIDH1 glioma. The risk model may act as an independent prognostic factor for IDH1mt glioma. IDH1mt glioma tissues from patients with high-risk scores showed more infiltration of M1 and CD8 T cells than those from patients with low-risk scores. Moreover, TIDE analysis showed that immune checkpoint blockade(ICB) therapy was highly beneficial for IDH1mt patients with high-risk scores. The risk model showed specific potential to predict the prognosis of IDH1mt-glioma patients, as well as guide ICB, contributing to the diagnosis and therapy of IDH1mt-glioma patients.

## Introduction

Glioma is the most common cerebral tumor with a high mortality rate (1). Several treatment approaches, including surgery and radio-chemotherapy, do not produce optimal results, and the average survival time of patients is less than 15 months (2, 3). Glioma is a highly heterogeneous tumor with multiple genetic characteristics, including isocitrate dehydrogenase (IDH1) mutation, 1p/19q-deficiency, and O-6-methylguanine-DNA methyltransferase methylation (4). Isocitrate dehydrogenase 1 is a key enzyme involved in the tricarboxylic acid cycle. In the cytoplasm and mitochondria, wild-type IDH1 (wtIDH1) oxidizes and decarboxylates isocitrate to α-ketoglutarate (α-KG), which is involved in epigenetic regulation and DNA repair in an α-KG-dependent manner (5, 6). A total of 70–80% of grade II and III gliomas and 80–90% of grade IV gliomas (also called glioblastomas) possess IDH1 mutations (IDH1mt) (7). Compared with glioma cells with wtIDH1, a hypermethylation phenotype, overactivated hypoxia signaling, and disruption of collagen maturation were observed in cells with IDH1mt (8). This difference emphasizes that different therapeutic strategies should be implemented for gliomas with wtIDH1 and IDH1mt mutations. The identification of specific biomarkers for gliomas with IDH1mt may contribute to this therapy.

Bioinformatics analysis using public databases, including The Cancer Genome Atlas (TCGA) and Gene Expression Omnibus, is a popular method for identifying tumor biomarkers (9, 10). Various biomarkers and risk models for gliomas have been identified through bioinformatic analysis. For example, by performing bioinformatics analysis in TCGA, the thioredoxin domain containing 11 was discovered to be upregulated in glioma tissues, and its high expression indicates a poor prognosis (11). An iron metabolism-related gene signature, constructed using a bioinformatics method, demonstrates that the risk model had remarkable prognostic value for gliomas (12). Similarly, by performing weighted gene co-expression analysis, our previous study indicated that LIM homeobox 5 and T-cell leukemia homeobox 1 are involved in the recurrence of glioma (13). However, the feasibility of biomarkers and risk models identified in previous studies for each subtype of glioma is limited.

Our study aimed to construct a specific risk model for predicting the prognosis of IDH1mt-glioma tissues and investigate the internal immunological and molecular mechanisms. Our risk model may provide insights into the diagnosis and treatment of IDH1mt gliomas.

## Materials and methods

### Gene expression profile download and preprocessing

The gene expression profiles of glioma patients and clinical trait information were downloaded from TCGA (https://portal.gdc.cancer.gov/) and the Chinese Glioma Genome Atlas (CGGA; http://www.cgga.org.cn/), respectively. The original gene expression profile was normalized and centralized, and the probe names were annotated as gene names. Before analysis, glioma patients without IDH1mt information and survival information were excluded. As a result, 367 patients with IDH1mt and 229 patients with wtIDH1 were obtained from TCGA, while 167 patients with IDH1mt and 145 patients with wtIDH1 were obtained from the CGGA. Immune and stromal scores of IDH1mt glioma tissues in TCGA were measured using the ESTIMATE algorithm.

### Differentially expressed genes analysis

The median immune and stromal scores of gliomas with IDH1mt in TCGA were used to separate the high and low groups. The EdgeR package was used to perform the differentially expressed genes (DEGs) analysis, while the threshold for considering significance was set as |logFC | ≥ 1 and adjusted *P*-value < 0.05. Analysis of the changes in all genes in the high- and low-immune/stromal score groups was visualized using volcano plots, while DEGs were visualized in a heatmap.

### Protein–protein interaction network

The primordial protein–protein interaction (PPI) network was constructed using DEG information from the STRING database (https://cn.string-db.org/). Cytoscape software was used to adjust the primordial PPI network, and the isolated genes were removed. Genes that had a relationship with others were set as hub genes and were enrolled in further studies.

### Enrichment analysis

The enriched GO terms of hub genes were analyzed using the Database for Annotation, Visualization, and Integrated Discovery (DAVID; https://david.ncifcrf.gov/). GO analysis was conducted using three categories: biological processes (BP), cellular components (CC), and molecular functions (MF). The significance threshold was set at *P* < 0.05. The top five terms are presented in a bubble diagram.

### Construction and verification of an immune signature

Before constructing an immune signature, we first analyzed the hub genes associated with the survival of patients with IDH1mt *via* univariate Cox regression analysis, and the significance threshold was set at *P* < 0.05. The least absolute shrinkage and selection operator (LASSO) was used to eliminate genes that shared similar genetic information by adding appropriate penalties (lambda). Utilizing the Akaike information criterion, an optimal prognostic risk model was built using a multivariate Cox regression analysis. The upper limit of the risk score was set to 10. The feasibility of the risk model in IDH1mt and wtIDH1 glioma patients in TCGA and CGGA was checked using receiver operator characteristic curve (ROC) analysis and Kaplan–Meier survival analysis. The ROC cut-off was set as the area under the curve (AUC) ≥ 0.75 and *P* < 0.05, while the threshold in the Kaplan–Meier survival analysis was set as *P* < 0.05.

### Nomogram construction

Nomograms are constructed based on multifactor regression analysis by integrating multiple predictors, and graduated line segments are then used to draw on the same plane in a certain proportion (14). In this study, a nomogram was created using the “rms” package to simplify the prediction model using independent clinical prognostic factors.

### Gene set enrichment analysis

To explore the signaling pathways differentially activated in high- and low-risk group IDH1mt-glioma patients in TCGA and CGGA, we analyzed the change of genes and performed GSEA analysis in R software with an adjusted *P*-value < 0.05.

### Immune cell analysis

Using the “CIBERSORT” R package, we examined 22 immune cells infiltrating IDH1mt-glioma tissues in TCGA and CGGA. Differentially infiltrated cells in the high- and low-risk-score groups were analyzed using the unpaired *t*-test, with significance set as *P* < 0.05.

### Immunohistochemical analysis

In total, 54 glioma tissues with IDH1mt were collected from the Affiliated Hospital of Guizhou Medical University with the approval of the Human Ethics Committee of Guizhou Medical University. None of the patients received any radio-chemotherapy before the operation, and written informed consent was obtained from all participants. Immunohistochemistry (IHC) staining was performed as described in our previous study (15). Primary antibodies used were as follows: HLA-DQA2 (1:200; Cat No. 42-669, ProSci, Fort Collins, CO, USA), HOXA3 (1:100; Cat No.ab230879, Abcam, Cambridge, UK), SAA2 (1:500; Cat No. CAU25292; Biomatik, Kitchener, Canada), CD8 (1:4000; Cat No. 66868-1-Ig; Proteintech, Wuhan, China), and CD86 (1:250; Cat No. ab220188, Abcam). The expression of target proteins was assessed based on the product of the intensity of staining (0, 1+, 2+, and 3+) and the percentage of positive cells, which was scored as 0 (0%), 1 (1–2 5%), 2 (26–50%), 3 (51–75%), or 4 (76–100%).

### T-cell dysfunction and exclusion analysis

To predict the immune checkpoint blockade (ICB) therapy response, the gene expression profile of glioma tissues with IDH1mt was imported into the T-cell dysfunction and exclusion (TIDE; http://tide.dfci.harvard.edu/) online algorithm to obtain exclusion, dysregulation, and TIDE scores. *P* < 0.05 was set as the threshold for determining the difference between high- and low-risk-score groups using an unpaired *t*-test.

## Results

### Landscape in high- and low-stromal/immune-score group glioma patients with IDH1mt in TCGA

**Figure 1:**
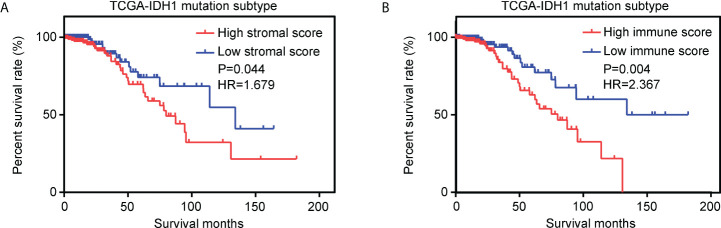
Effects of stromal and immune scores on the survival of IDH1mt-glioma patients. **(A)** Kaplan–Meier survival analysis showing the survival rate in high- and low-stromal-score-group IDH1mt-glioma patients. **(B)** Kaplan–Meier survival analysis showing the survival rate in high- and low-immune-score-group IDH1mt-glioma patients.

**Figure 2:**
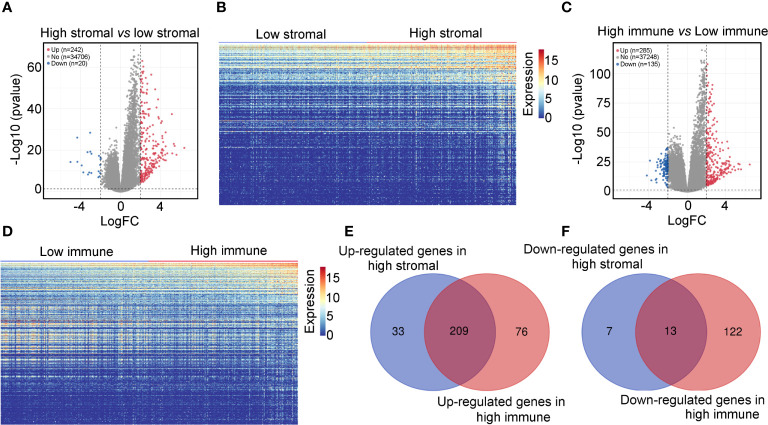
Differentially expressed genes in high- and low-stromal/immune-score group IDH1mt-glioma tissues. **(A)** Volcano plot demonstrating the differential expression of genes in high- and low-stromal group of IDH1mt-glioma tissues. **(B)** Heatmap plot showing DEGs between high- and low-stromal group of IDH1mt-glioma tissues. **(C)** Volcano plot demonstrating the differential expression of genes in high- and low-immune group of IDH1mt-glioma tissues. **(D)** Heatmap plot showing DEGs between high- and low-immune group of IDH1mt-glioma tissues. **(E)** Overlapping upregulated genes between high-stromal and -immune group of IDH1mt-glioma tissues. **(F)** Overlapping downregulated genes between high-stromal and -immune group of IDH1mt-glioma tissues.

Using the ESTIMATE algorithm, stromal and immune scores were calculated in TCGA for patients with glioma and IDH1mt. The detailed scores are shown in **Supplementary Table 1**. The Kaplan–Meier survival analysis showed that IDH1mt-glioma patients with high stromal (**Figure 1A**) and immune scores (**Figure 1B**) exhibited shorter overall survival rates than those with low stromal and immune scores (HR = 1.679 and HR = 2.367). We then determined the change in gene expression profiles between the high- and low-stromal/immune groups in IDH1mt-glioma tissues. A total of 242 genes were upregulated in the high-stromal group IDH1mt-glioma tissues compared with those in the low-stromal group IDH1mt-glioma tissues, while 20 genes were downregulated (**Figures 2A, B**). Similarly, 285 genes were upregulated in the high-immune group IDH1mt-glioma tissues compared with those in the low-immune group IDH1mt-glioma tissues, while 135 genes were downregulated (**Figures 2C, D**). Through intersection analysis, 209 upregulated (**Figure 2E**) and 13 downregulated overlapping genes (**Figure 2F**) were identified in the high-stromal- and high-immune-score-group IDH1mt-glioma tissues.

**Figure 3:**
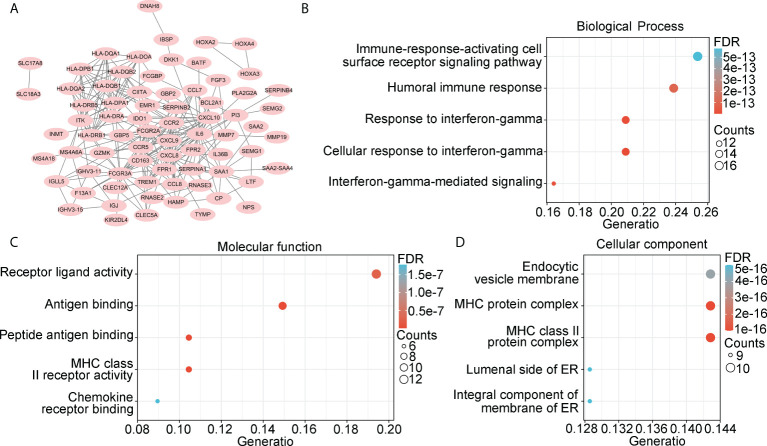
Landscape of 222 overlapping DEGs. **(A)** PPI network was constructed using 222 overlapping DEGs, while isolated genes were removed. Genes in PPI network were set as candidate hub genes. **(B)** Biological process (BP) analysis for candidate hub genes. **(C)** Molecular function (MF) analysis for candidate hub genes. **(D)** Cellular component (CC) analysis for candidate hub genes.

We then constructed a PPI network and found 72 genes that interacted with other genes (**Figure 3A**). These genes were set as candidate hub genes associated with stromal and immune scores in IDH1mt glioma. BP enrichment analysis demonstrated that these candidate hub genes were enriched in “immune-response-activating cell surface receptor signaling pathway”, “humoral immune response”, “response to interferon-gamma”, “cellular response to interferon gamma”, and “interferon-gamma-mediated signaling” (**Figure 3B**). MF enrichment analysis demonstrated that the candidate hub genes were enriched in “receptor ligand activity”, “antigen binding”, “peptide antigen binding”, “MHC class II receptor activity”, and “chemokine receptor binding” (**Figure 3C**). Furthermore, the enriched CC terms of candidate hub genes were “endocytic versicle membrane”, “MHC protein complex”, “MHC class II protein complex”, “luminal side of ER”, and “integral component of membrane of ER” (**Figure 3D**).

### Construction of immune signature for glioma patients with IDH1mt

**Figure 4:**
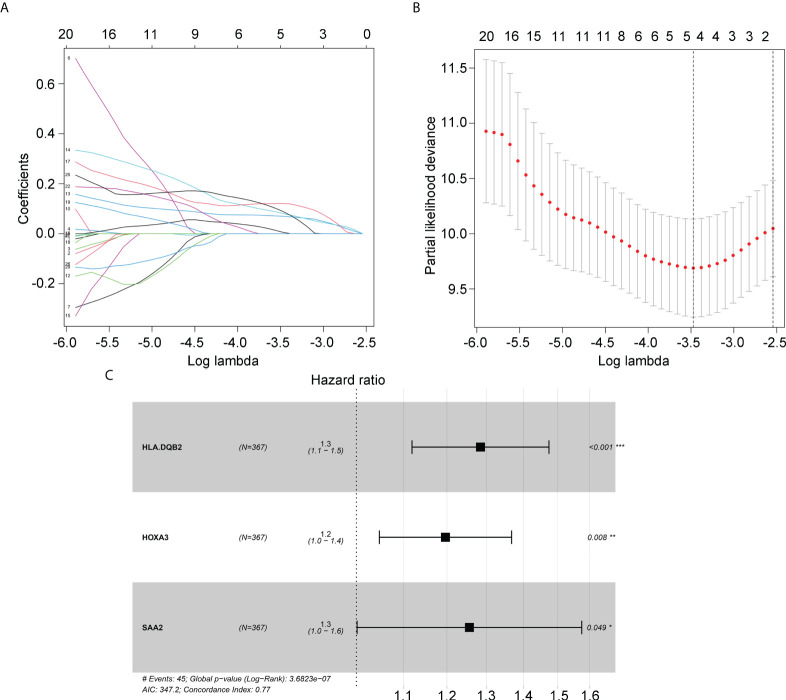
Hub genes selected to construct the risk model. **(A, B)** LASSO analysis for hub genes associated with the survival rate of IDH1mt-glioma patients. **(C)** Multivariate Cox regression analysis of *HLA-DQA2*, *HOXA3*, and *SAA2.* These three genes were used to construct the risk model.

Next, we determined whether the expression of candidate hub genes was associated with survival in patients with IDH1mt gliomas. A univariate Cox regression analysis demonstrated that among 72 candidate hub genes, 29 genes were linked to survival in IDH1mt-glioma patients (**Table 1**). LASSO analysis was performed to create a risk model that could predict the survival of glioma patients with IDH1mt. Five more important hub genes, including *HLA-DQA2*, *HLA-DQB2*, *HOXA2*, *HOXA3*, and *SAA2*, were identified and used for further analysis (**Figures 4A, B**). A multivariate Cox analysis also indicated that *HLA-DQA2*, *HOXA3*, and *SAA2* were independent predictors of survival in glioma patients with IDH1mt (**Figure 4C**). Therefore, the gene expression information of *HLA-DQA2*, *HOXA3*, and *SAA2* and the survival information of glioma patients with IDH1mt in TCGA were imported into R software to construct a risk model. Using computer optimization, a risk model was constructed with a risk score of 0.249 × *HLA-DQA2* expression + 0.179 × *HOXA3* expression + 0.227 × *SAA2* expression.

**Table 1:** The hazard rate of genes for glioma patients with IDH1mt.

ID	HR	HR.95L	HR.95H	pvalue
BATF	1.17470829	0.955519886	1.444176707	0.126474997
BCL2A1	1.07719203	0.903329599	1.284517483	0.407704967
CCL7	0.97309829	0.69982352	1.353084395	0.871200368
CCL8	1.04917291	0.926433995	1.18817293	0.449528605
CCR2	1.12311681	0.944634881	1.335321607	0.188534973
CCR5	1.22830925	1.020308646	1.478712945	0.029828985
CD163	1.1776415	1.031132277	1.344967592	0.01585487
CIITA	1.28208779	1.054369628	1.558987519	0.012752323
CLEC12A	1.16773723	0.987945732	1.380248118	0.069094998
CLEC5A	1.126091	0.982655187	1.290463802	0.087586721
CP	1.21627375	1.048854918	1.410416063	0.009562912
CXCL10	1.14573922	1.000603026	1.311927238	0.048989007
CXCL8	1.02105507	0.903983423	1.153288238	0.737363758
CXCL9	1.10750092	0.938818303	1.306491669	0.225850624
DKK1	0.99466229	0.8374469	1.181392011	0.951382525
DNAH8	1.01804052	0.808774932	1.28145231	0.878959919
F13A1	0.99685455	0.841916679	1.180305617	0.970841318
FCGBP	1.10629314	0.961207373	1.27327832	0.159028039
FCGR2A	1.41700439	1.136628579	1.766541396	0.001945538
FCGR3A	1.21004902	1.018480893	1.437649593	0.030142571
FGF3	1.20176804	0.756735262	1.908522706	0.436089198
FPR1	1.13504686	0.960628616	1.341133659	0.136724904
FPR2	1.17963747	0.993020217	1.401325519	0.060074354
GBP2	1.1632864	0.965164204	1.402077744	0.112342727
GBP5	1.3123034	1.091287426	1.57808125	0.003872657
GZMK	1.12873693	0.939006147	1.356803755	0.19714833
HAMP	1.07895365	0.939439217	1.239187127	0.282076153
HLA.DOA	1.24287069	1.044807964	1.478479885	0.014093502
HLA.DPA1	1.31348349	1.095910008	1.574252317	0.00316582
HLA.DPB1	1.28704422	1.069243207	1.549210518	0.007636075
HLA.DQA1	1.17474627	1.013513368	1.361628609	0.032502989
HLA.DQA2	1.28594457	1.146262918	1.442647596	1.81E-05
HLA.DQB1	1.11087796	0.947045636	1.303052149	0.196483662
HLA.DQB2	1.35395048	1.180491115	1.552897666	1.48E-05
HLA.DRA	1.2694638	1.065062313	1.513092999	0.007729404
HLA.DRB1	1.20054844	0.999966103	1.441365415	0.050042495
HLA.DRB5	1.11853935	0.947546333	1.320389541	0.185690004
HOXA2	1.32415251	1.161028243	1.5101957	2.84E-05
HOXA3	1.27018869	1.137417785	1.418457955	2.18E-05
HOXA4	1.33711113	1.161375341	1.539438726	5.32E-05
IBSP	1.09171415	0.975610656	1.221634661	0.126125388
IDO1	1.11442618	0.971509083	1.278367576	0.121819576
IGHV3.11	1.15002804	1.001887773	1.320072493	0.046947954
IGHV3.15	1.10670447	0.930408719	1.316405095	0.252122931
IGLL5	1.09127324	0.973389831	1.223433038	0.134250793
IL36B	1.45331441	1.121085057	1.883998699	0.00475667
IL6	1.02151333	0.901654664	1.157305041	0.73818588
INMT	0.98625239	0.824692471	1.179462421	0.879457272
ITK	1.13922357	0.943975874	1.374855413	0.174178403
KIR2DL4	1.10475317	0.886294397	1.377058872	0.375508073
LTF	1.07336712	0.963535322	1.195718473	0.198614155
MMP19	1.07573521	0.906241704	1.276928928	0.403973391
MMP7	1.08890983	0.9733895	1.218139933	0.136591778
MS4A18	1.16103337	0.87338476	1.54341882	0.303979155
MS4A6A	1.30235412	1.076392497	1.575750711	0.006585199
NPS	1.30028934	1.038978688	1.627321513	0.021789224
PI3	1.11206459	0.970938203	1.273703776	0.125022883
PLA2G2A	1.07054961	0.956105386	1.198692621	0.237279756
RNASE2	1.20247034	1.027392368	1.40738335	0.021643479
RNASE3	1.13588087	0.947471011	1.361757073	0.168558144
SAA1	1.27770512	1.092172861	1.494754574	0.002203186
SAA2	1.54587115	1.253874467	1.905866717	4.54E-05
SAA2.SAA4	1.71834957	1.295116327	2.27989192	0.000175121
SEMG1	0.73695081	0.422560476	1.285251517	0.282096946
SEMG2	0.81504085	0.440134346	1.509292788	0.515332513
SERPINA1	1.23919535	1.025166407	1.497908151	0.026630699
SERPINB2	0.97940182	0.837863545	1.144849821	0.793822582
SERPINB4	0.74079768	0.449792849	1.220075431	0.238563585
SLC17A8	0.97157479	0.873003646	1.081275643	0.597273097
SLC18A3	1.17226028	1.013940044	1.355301202	0.031795339
TREM1	1.12228451	0.978805459	1.286795553	0.098327687
TYMP	1.25469942	1.045712163	1.505453117	0.01465489

### Verification of applicability of risk model in glioma patients with IDH1mt in TCGA and CGGA

**Figure 5:**
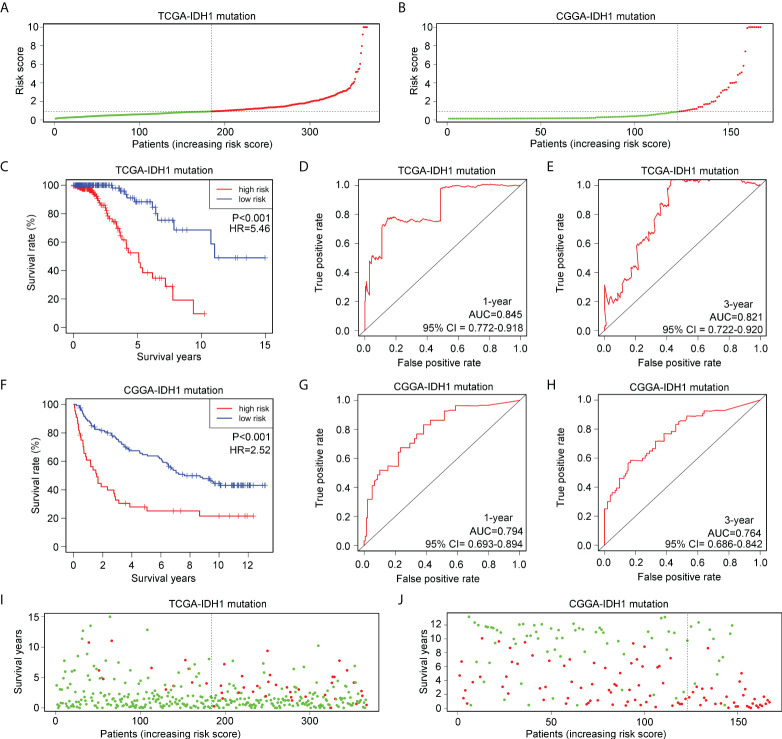
Verification of the applicability of the risk model in IDH1mt-glioma patients in TCGA and CGGA databases. **(A, B)** IDH1mt-glioma patients in TCGA and CGGA databases were divided into high- and low-risk score groups according to the median of risk scores. **(C)** The survival difference between high- and low-risk score group IDH1mt-glioma patients in TCGA. **(D, E)** The diagnostic value of risk model for one- and three-year survival in IDH1mt-glioma patients in TCGA. **(F)** The survival difference between high- and low-risk score group IDH1mt-glioma patients in CGGA. **(G, H)** The diagnostic value of risk model for one- and three-year survival in IDH1-mt glioma patients in CGGA. **(I, J)** Death cases in high- and low-risk score group IDH1mt-glioma patients in TCGA and CGGA (Green dots mean alive cases, red dots mean death cases).

To verify the applicability of the risk model in glioma patients with IDH1mt, glioma patients with IDH1mt in TCGA (**Figure 5A**) and CGGA (**Figure 5B**) were divided into high- and low-risk groups according to the median risk score obtained from the TCGA cohort. The results indicated that IDH1mt glioma patients in TCGA with high-risk scores had shorter overall survival rates than those with low-risk scores (**Figure 5C**). ROC analysis indicated that the AUC for predicting the one- and three-year survival of IDH1mt-glioma patients in TCGA were 0.845 and 0.821, respectively (**Figures 5D, E**). Similarly, we found that IDH1mt-glioma patients in CGGA with high-risk scores had shorter overall survival rates than those with low-risk scores (**Figure 5F**), and the AUC for predicting one- and three-year survival of IDH1mt-glioma patients in CGGA were 0.794 and 0.764, respectively (**Figures 5G, H**). Furthermore, we found that IDH1mt-glioma patients in TCGA (**Figure 5I**) and CGGA (**Figure 5J**) with high-risk scores exhibited a higher proportion of deaths. This evidence indicates that the risk model has remarkable diagnostic value for glioma patients with IDH1mt.

### Exploration of applicability of risk model in glioma patients with wtIDH1 in TCGA and CGGA

**Figure 6:**
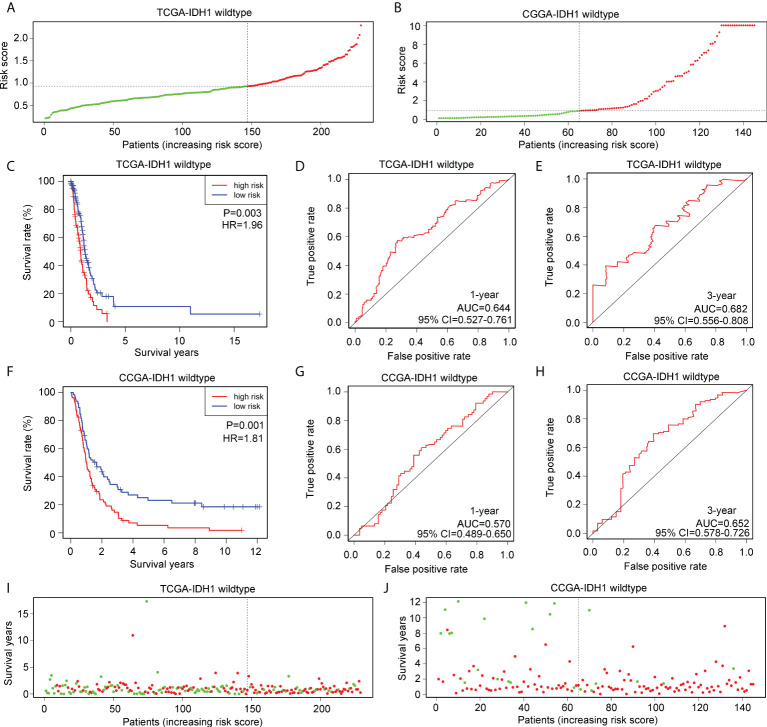
Verification of the applicability of the risk model in wtIDH1-glioma patients in TCGA and CGGA databases. **(A, B)** wtIDH1-glioma patients in TCGA and CGGA databases were divided into high- and low-risk score groups according to the median of risk scores. **(C)** The survival difference between high- and low-risk score group wtIDH1-glioma patients in TCGA. **(D, E)** The diagnostic value of risk model for one- and three-year survival in wtIDH1-glioma patients in TCGA. **(F)** The survival difference between high- and low-risk score group wtIDH1-glioma patients in CGGA. **(G, H)** The diagnostic value of the risk model for one- and three-year survival in wtIDH1-glioma patients in CGGA. **(I, J)** Death cases in high- and low-risk score group wtIDH1-glioma patients in TCGA and CGGA (Green dots mean alive cases, red dots mean death cases).

Glioma patients with wtIDH1 in TCGA (**Figure 6A**) and CGGA (**Figure 6B**) were divided into high- and low-risk groups according to the median risk score. wtIDH1 glioma patients in TCGA with high-risk scores had lower overall survival than those with low-risk scores (**Figure 6C**). However, the AUC for predicting the one- and three-year survival of wtIDH1 glioma patients in TCGA were 0.644 and 0.682, respectively (**Figures 6D, E**). Similarly, high-risk wtIDH1-glioma patients in CGGA had shorter overall survival rates (**Figure 6F**), but the AUCs of the risk model for predicting the one- and three-year survival of wtIDH1 glioma patients in CGGA were 0.570 and 0.652, respectively (**Figures 6G, H**). Furthermore, the percentage of deaths was not significantly different between wtIDH1 glioma patients in the high- and low-risk groups in TCGA (**Figure 6I**) and CGGA (**Figure 6J**). These results indicated that the risk model constructed using *HLA-DQA2*, *HOXA3*, and *SAA2* was not suitable for predicting the survival of wtIDH1 glioma patients and may be specific for IDH1mt-glioma patients.

### Immune signature acts as independent prognostic factor for glioma patients with IDH1mt

**Figure 7:**
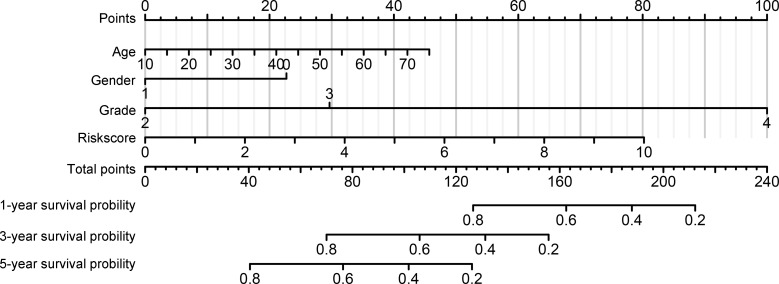
Construction of nomogram based on the signature risk score and clinical characteristics.

We then performed a multivariate Cox regression analysis, and the immune signature constructed using *HLA-DQA2*, *HOXA3*, and *SAA2* was found to act as an independent prognostic factor for glioma patients with IDH1mt, with an HR of 1.203 (**Table 2**). In addition, a nomogram was created based on the signature risk score and clinical characteristics (**Figure 7**).

**Table 2:** Cox regression analysis of the immune signature.

Characteristics	HR (95% CI) Univariate analysis	P value Univariate analysis	HR (95% CI) Multivariate analysis	P value Multivariate analysis
Age	1.017 (1.001-1.034)	0.042	1.016 (1.000-1.033)	0.048
Gender				
0				
1	0.744 (0.534-1.036)	0.049	0.592 (0.421-0.834)	0.003
Grade				
2				
3	2.280 (1.532-3.394)	<0.001	1.981 (1.316-2.982)	0.001
4	12.863 (8.264-20.022)	<0.001	10.030 (6.184-16.268)	<0.001
riskScore	1.382 (1.286-1.485)	<0.001	1.203 (1.113-1.300)	<0.001

### Exploration of pathways associated with immune signature

**Figure 8:**
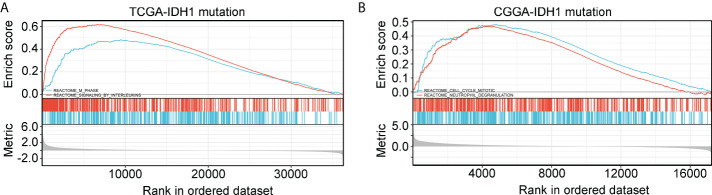
GSEA analysis of the pathway terms enriched in high-risk score IDH1mt-glioma tissues in TCGA **(A)** and CGGA **(B)**.

Gene set enrichment analysis (GSEA) was used to determine if defined pathways were enriched in high- and low-risk groups of glioma patients with IDH1mt. IDH1mt-glioma tissues with high risk in TCGA were positively associated with “M phase“ (NES=1.89, P<0.01) and “signaling by interleukins” (NES=2.24, P<0.01; **Figure 8A**), while those with high risk in CGGA were positively associated with “cell cycle mitotic” (NES=2.19, P<0.01) and “neutrophil degranulation” (NES=2.17, P<0.01; **Figure 8B**).

### Immune characteristics of the immune signature

**Figure 9:**
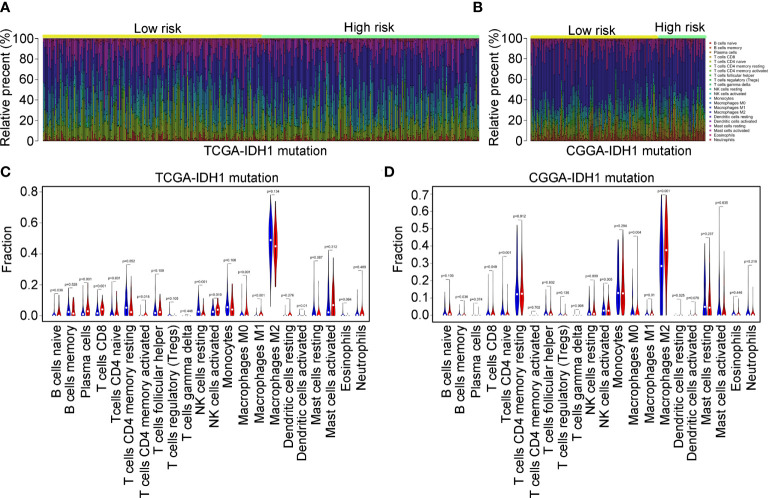
Immune characteristics of the three immune signatures. **(A, B)** The gene expression profiles of the high- and low-risk score group IDH1mt-glioma tissues in TCGA and CGGA were converted into 22 immune cell expression matrices. **(C, D)** Difference in immune cells between high- and low-risk score group IDH1mt-glioma tissues in TCGA and CGGA.

According to previous studies (16, 17), infiltrating immune cells play a critical role in the progression of IDH1mt glioma. We determined the difference in infiltration of 22 immune cells between the high- and low-risk groups of glioma tissues with IDH1mt. The CIBERSORT R package was used to convert the gene expression profile of glioma tissues with IDH1mt in TCGA (**Figure 9A**) and CGGA (**Figure 9B**) to a proportion profile of infiltrated immune cells. Compared with IDH1mt glioma tissues with low-risk scores, those with high-risk scores in TCGA exhibited a high proportion of naïve B cells, plasma cells, CD8 T cells, CD4 memory activated T cells, activated NK cells, M0 macrophages, and M1 macrophages, while the proportion of resting NK cells and activated dendritic cells was reduced (**Figure 9C**). In CGGA, IDH1mt-glioma tissues with high-risk scores had higher memory B cells, CD8 T cells, M1 macrophages, M2 macrophages, and resting dendritic cells and lower M0 and CD4 naïve T cells (**Figure 9D**). In conclusion, both TCGA and CGGA indicated a higher proportion of CD8 T cells and M1 macrophages in IDH1mt-glioma tissues with high risk compared with that in those with low risk.

### Detection of the expression of *HLA-DQA2, HOXA3, SAA2, CD8*, and *CD86* in IDH1mt-glioma tissues

**Figure 10:**
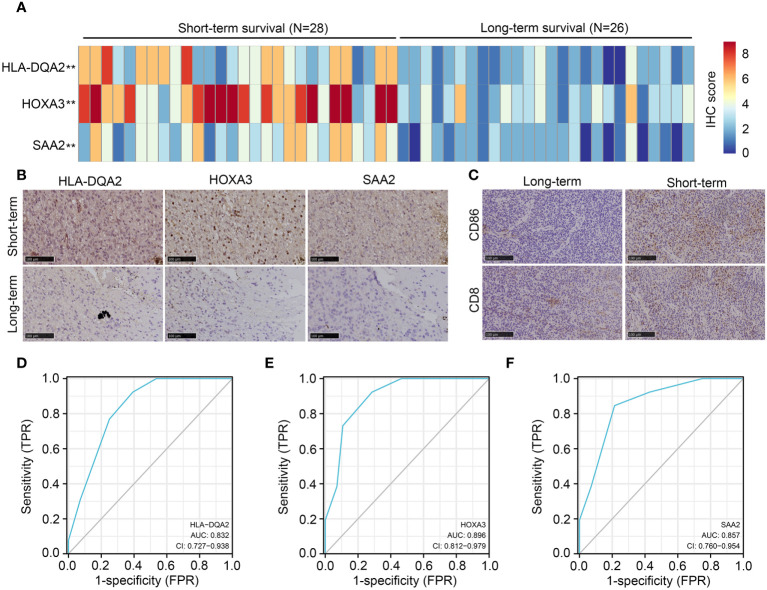
Expression of *HLA-DQA2*, *HOXA3*, *SAA2*, *CD86*, and *CD8* in IDH1mt-glioma tissues. IDH1mt-glioma tissues were divided into long- and short-term survival groups according to the patient’s number of days of survival with the cut-off as 15 months. **(A)** The IHC score of *HLA-DQA2*, *HOXA3*, and *SAA2* in IDH1mt-glioma tissues in long- and short-term groups. **(B)** Representative figures of expression of *HLA-DQA2*, *HOXA3*, and *SAA2* in long- and short-term group IDH1mt-glioma tissues. **(C)** Expression of *CD86* and *CD8* in long- and short-term group IDH1mt-glioma tissues. **(D–F)** The diagnostic value of *HLA-DQA2*, *HOXA3*, and *SAA2* for distinguishing long- and short-term survival of IDH1mt-glioma patients. **P < 0.01.

In total, 54 glioma tissues with IDH1mt from our research group were divided into long- (survival ≥ 15 months) and short-term groups (survival < 15 months). IHC was performed to detect the expression of *HLA-DQA2*, *HOXA3*, and *SAA2* in glioma tissues, and high expression of *HLA-DQA2*, *HOXA3*, and *SAA2* was observed in glioma tissues in the short-term group compared with that in the long-term group (**Figures 10A, B**). Similarly, we detected the expression of the M1 biomarker *CD86* and the CD8 T-cell biomarker *CD8* in glioma tissues using IHC. The expression of *CD86* and *CD8* increased in IDH1mt-glioma tissues in the short-term survival group (**Figure 10C**). These results suggest that M1 and CD8 T cells infiltrate more deeply into IDH1mt-glioma tissues associated with a lower probability of survival (**Figure 10C**). Furthermore, ROC analysis was performed to determine the diagnostic value of *HLA-DQA2*, *HOXA3*, and *SAA2* in the survival of IDH1mt-glioma patients, and all showed remarkable diagnostic value (AUC = 0.832, 0.896, and 0.857) (**Figures 10D–F**).

### Glioma patients with IDH1mt in high-risk group exhibited high responsiveness to ICB therapy

**Figure 11:**
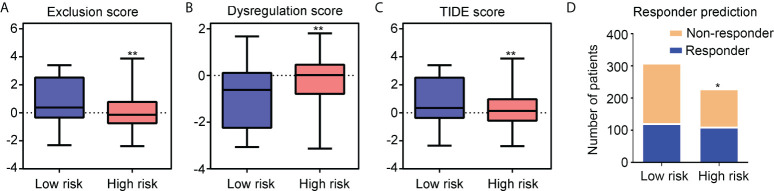
Glioma patients with IDH1mt in high-risk group exhibit high responsiveness to ICB therapy. **(A)** Exclusion score of glioma patients with IDH1mt in high- and low-risk groups. **(B)** Dysregulation score of glioma patients with IDH1mt in high- and low-risk groups. **(C)** TIDE score of glioma patients with IDH1mt in high- and low-risk groups. **(D)** Responders and non-responders among glioma patients with IDH1mt in high- and low-risk groups. *P < 0.05; **P < 0.01.

The TIDE online algorithm was used to evaluate the responsiveness of IDH1mt-positive glioma patients in the high- and low-risk groups to ICB therapy. Lower exclusion scores were observed in IDH1mt-glioma patients with high-risk scores than in those with low-risk scores (**Figure 11A**), while the dysregulation score was reduced (**Figure 11B**). Overall, the TIDE score was significantly reduced in IDH1mt-glioma tissues with high-risk scores compared with that in those with low-risk scores (**Figure 11C**). Finally, the responder prediction results indicated that glioma patients with IDH1mt in the high-risk group exhibited high responsiveness to ICB therapy (**Figure 11D**). Based on this evidence, this risk model may be able to guide the clinical treatment of glioma patients with IDH1mt.

## Discussion

Among primary brain tumors, malignant gliomas are the most common and show a poor prognosis (18). One of the most common genetic lesions in gliomas is a heterozygous mutation in IDH1, which occurs in 70–80% of grade II or III gliomas and most secondary glioblastomas (7). IDH1mt induces high histone methylation, high DNA methylation, high DNA damage response, and low amino acid metabolism in glioma cells (8). Due to the specific molecular mechanisms involved in the progression of glioma with IDH1mt, some biomarkers and therapeutic drugs may not be suitable for the IDH1mt subtype. The identification of specific biomarkers for gliomas with IDH1mt may aid diagnosis and therapy.

As previous studies have indicated that dysregulation of immune microenvironments is involved in the progression of gliomas with IDH1mt (19, 20), we first calculated the stromal and immune scores in glioma tissues with IDH1mt. We found that IDH1mt-glioma patients with high stromal/immune scores had lower survival rates than those with low stromal/immune scores. We then focused on the DEGs between the high and low stromal/immune score groups of IDH1mt-glioma. In total, 222 DEGs were identified, while 29 genes interacted with others in the PPI network and were significantly associated with prognosis. Then, *via* LASSO and Cox regression analyses, immune signatures were constructed using *HLA-DQA2*, *HOXA3*, and *SAA2*, and IDH1mt-glioma patients were divided into high-risk and low-risk groups. Risk models have been constructed for gliomas and exhibited remarkable prognostic value (21, 22). However, the prognostic value of these risk models for each subtype of glioma is limited, which restricts their clinical application.

*HLA-DQA2* belongs to the HLA class II alpha chain family, and its encoded protein forms a heterodimer with a class II beta chain, contributing to the present antigenic peptides (23). Previous studies indicated that *HLA-DQA2* mutations were associated with the susceptibility of lung cancer (24). However, its role in glioma was still known limit. *HOXA3* encodes a DNA-binding transcription factor, which involved in the embryonic development through regulating genes of morphogenesis and cell differentiation (25). Upregulated HOXA3 was observed in series of cancers, including glioma (26). SAA2 encodes a member of the serum amyloid A family of apolipoproteins, which would elevated in the tissues with inflammation (27). *SAA2* encoded protein plays an important role in HDL metabolism and cholesterol homeostasis (28). Previous studies indicated that high level of *SAA2* was associated with the progression of inflammatory disease, including cancer (29). In glioma, high expression of SAA2 was associated with temozolomide resistance (30). In this study, we focused on the IDH1mt subtype glioma and found that the risk model constructed using *HLA-DQA2*, *HOXA3*, and *SAA2* showed remarkable prognostic value for IDH1mt glioma in both TCGA and CGGA cohorts but not for wtIDH1-glioma. Furthermore, this risk model may act as an independent prognostic factor for IDH1mt glioma. We suggest that this risk model constructed using immune-related genes may characteristically contribute to the assessment of the prognosis of IDH1mt glioma.

The tumor environment (TME) is a complex integrated system that contains cancer cells, immune cells, inflammatory cells, tumor-associated fibroblasts, and various cytokines (31, 32). Immune cells infiltrating the TME participate in the progression of glioma. For example, high number of cells are polarized to M2 phenotype in glioma tissues and have the potential to enhance the invasiveness of glioma cells by inducing angiogenesis, whereas M1 cells have the opposite effects (33). NK and CD8 T cells have the potential to induce senescence in glioma cells (34). However, the immune signature of IDH1mt glioma is limited. In this study, we found that high levels of M1 and CD8 T cells were more prevalent in IDH1mt patients with high-risk scores in both the TCGA and CGGA cohorts. Regarding the cancer-killing effects of M1 and CD8 T cells, lower survival rates were observed in IDH1mt-glioma patients with high-risk scores and high M1 and CD8 T cells infiltration. To explore the mechanism, TIDE was performed, and we found that IDH1mt-glioma patients with high-risk scores had high dysregulation scores and low exclusion scores. This evidence suggests that the TME of IDH1mt-glioma patients with high-risk scores may inhibit the functions of M1 and CD8 T cells and that they cannot exert their function, even though they show high infiltration.

ICB is a potential anti-tumor therapy that exhibits significant curative effects in a range of cancer types, including hepatocellular carcinoma (35) and breast cancer (36). By blocking immune checkpoints, deactivated cells can be reactivated to help the host kill cancer cells (37, 38). However, evidence of the benefits of ICB in gliomas with IDH1mt is limited. As evidenced that TME in IDH1mt-glioma patients in the high-risk score group can induce the inactivation of M1 cells and CD8 T cells, we furthered analyzed whether ICB had a high benefit for IDH1mt-glioma patients in the high-risk score group. Compared with those in the low-risk score group, the TIDE score and response rate of ICB were higher in the high-risk score group. This indicates that ICB may improve the prognosis of IDH1mt-glioma patients with high-risk scores.

However, there are some limitations in our present study. Compared with the samples in TCGA and CGGA, the samples from our research group is quite little. Furthermore, more experiments should be performed to determine how *HLA-DQA2*, *HOXA3*, and *SAA2* affect the TME.

In conclusion, an immune signature constructed using *HLA-DQA2*, *HOXA3*, and *SAA2* exhibited significant and specific prognostic value for IDH1mt glioma, while the high-risk group classified by the signature had a high benefit from ICB. This immune signature may contribute to the diagnosis and treatment of IDH1-mt gliomas.

## Data availability statement

The datasets presented in this study can be found in online repositories. The names of the repository/repositories and accession number(s) can be found in the article/**Supplementary Material**.

## Ethics statement

Written informed consent was obtained from the individual(s) for the publication of any potentially identifiable images or data included in this article.

## Author contributions

PP, FP, and TC designed the experiments. ZZ, CH, and WR performed the analyses and parts of the experiments. SL, YY, and JZ performed experiments. All the authors have read and agreed to submit the final version of the manuscript. All authors contributed to the article and approved the submitted version.

## Funding

This research was supported by National Natural Science Foundation of China (Grant No. 82160665), the Project of Science and Technology Department of Guizhou Province [Qiankehe support (2021) general 089], Guizhou Provincial Health Commission project (No. gzwjkj2020-1-037), and the continuous support fund for excellent scientific research platform of colleges and universities in Guizhou Province (QJHRCTD [2015]51).

## Conflict of interest

The authors declare that the research was conducted in the absence of any commercial or financial relationships that could be construed as a potential conflict of interest.

## Publisher’s note

All claims expressed in this article are solely those of the authors and do not necessarily represent those of their affiliated organizations, or those of the publisher, the editors and the reviewers. Any product that may be evaluated in this article, or claim that may be made by its manufacturer, is not guaranteed or endorsed by the publisher.
